# Prognosis of tricuspid regurgitation after mitral transcatheter edge-to-edge repair: the EXPANDed studies

**DOI:** 10.1093/eschf/xvag108

**Published:** 2026-04-16

**Authors:** Rodrigo Estevez-Loureiro, Mark J Ricciardi, Wolfgang Rottbauer, Matthew J Price, Philip Raake, Mathew Williams, Federico M Asch, Jose L Zamorano, Melody Dong, Kelli Peterman, Evelio Rodriguez, Saibal Kar, Ralph Stephan von Bardeleben, Francesco Maisano

**Affiliations:** Department of Cardiology, University Hospital Álvaro Cunqueiro, Estrada de Clara Campoamor, 341, Vigo, Pontevedra 36213, Spain; Department of Cardiology, Endeavor Health/Northshore University Hospital, Chicago, IL, USA; Department of Cardiology, Ulm, Germany; Department of Cardiology, Scripps, La Jolla, CA, USA; Department of Cardiology, University of Heidelberg, Germany; Department of Cardiology, NYU, NewYork, NY, USA; Department of Cardiology, Medstar Health Research Institute, Washington, DC, USA; Department of Cardiology, Hospital Ramon y Cajal, Spain; Abbott Structural Heart, Santa Clara, CA, USA; Abbott Structural Heart, Santa Clara, CA, USA; Department of Cardiology, St.Thomas, Nashville, TN, USA; Department of Cardiovascular Surgery, Thousand Oaks, Los Robles, CA, USA; Department of Cardiology, Mainz, Germany; Department of Cardiovascular Surgery, Ospedale San Raffaele, Italy

**Keywords:** Mitral regurgitation, Tricuspid regurgitation, Transcatheter repair, Mitral valve repair, MitraClip

## Abstract

**Background:**

Transcatheter therapies offer new treatment options for patients with both mitral regurgitation (MR) and tricuspid regurgitation (TR). However, the optimal treatment pathway in patients with combined MR and TR is not completely understood.

**Aims:**

This analysis evaluated the natural TR progression after mitral transcatheter edge-to-edge repair (MTEER) with the MitraClip System in patients with MR and TR from the EXPANDed studies.

**Methods:**

EXPANDed is a pooled cohort from the EXPAND and EXPAND G4 studies. This study includes patients who had severe TR, achieved procedural success with MTEER, and received no direct TR intervention. Echocardiographic assessments were performed independently by echo core lab. Baseline characteristics, 1-year outcomes, and associations with TR improvement were reported based on 30-day TR severity following MTEER.

**Results:**

Of those with evaluable TR data at 30 days (*N* = 160), 73% (*N* = 116) improved to ≤moderate TR, while 28% (*N* = 44) had ≥severe TR. The ≤moderate TR group had a lower prevalence of atrial fibrillation (68% vs 89%, *P* = .009), numerically lower LV ejection fraction (49% vs 56%, *P* = .07), and larger LV dimensions (LVEDV: 137.5 ± 73.4 vs 107.9 ± 44.8 ml, *P* = .01). TR reduction was sustained in 86% of ≤moderate TR patients, while 45% of ≥severe TR patients improved to ≤moderate at 1 year. In the ≤moderate TR group, significant and larger improvements in NYHA functional class (*P* < .0001) and KCCQ-OS score (Δ = + 30.6 ± 25.7, *P* < .0001) were observed through 1 year. One-year mortality was numerically lower in the ≤moderate TR group (12.4% vs 22.3%) though not statistically significant (HR = 1.92 [.77, 4.79], *P* = .16). Lower LVEF and larger baseline LV size were associated with TR improvement post-MTEER.

**Conclusions:**

Early TR improvement to ≤moderate was observed in almost 3/4 of the population and was associated with significant symptomatic relief. Patients with both severe MR and TR, particularly those with LV dilation, may experience TR improvement following MTEER.

## Graphical Abstract

**Graphical Abstract:**
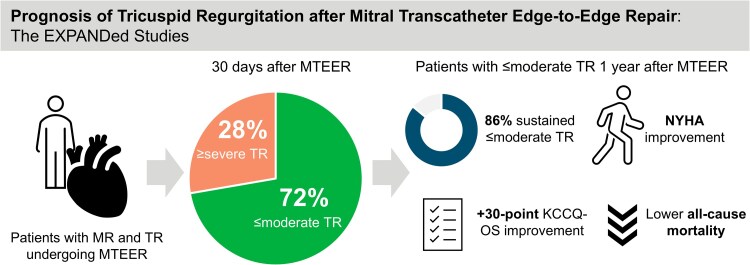

## Introduction

Mitral transcatheter edge-to-edge repair (MTEER) has become an established treatment for patients with mitral regurgitation (MR) regardless of aetiology.^1,2^ Tricuspid regurgitation (TR) is common in MR patients and may result from chronic pressure and volume overload transmitted backward from the left ventricle (LV) to the right heart chambers. Previous studies have found that 20%-30% of patients with MR have moderate or greater TR, which is associated with worse outcomes compared with isolated MR.^3,4^

Severity of baseline TR has been identified as a predictor of mortality and heart failure hospitalizations in patients with MR undergoing conventional surgery.^5^ Additionally, several studies have demonstrated the detrimental effect of baseline TR on outcomes of MTEER.^6–9^ Guidelines recommend an aggressive treatment approach to TR due to its tendency to progress after surgery and its negative impact on prognosis.^10,11^ Promising results from the TRILUMINATE Pivotal and TRISCEND II trials and the respective FDA approvals of the TriClip TEER device and EVOQUE tricuspid replacement valve have introduced a new treatment pathway for patients with severe TR.^12–14^ However, optimal management of patients with both MR and TR remain unclear. Some cases of TR may spontaneously resolve following MR reduction, while others may require targeted treatment.

Previous small-scale registries have explored TR after MTEER and identified potential predictors of secondary TR improvement.^7,15–17^ However, most of these studies were retrospective and lacked consistent assessments of TR by an independent echocardiographic core lab (ECL). This subgroup analysis from the global, post-market EXPANDed studies aimed to identify and evaluate the evolution and clinical impact of TR in patients with concomitant MR and TR using ECL-assessed measures after MTEER treatment with the MitraClip device.

## Methods

### Study design and analysis population

EXPANDed is a patient-level pooled cohort combining data from two global post-market studies: EXPAND (A Contemporary, Prospective Study Evaluating Real-world Experience of Performance and Safety for the Next Generation of MitraClip Devices; NCT03502811) and EXPAND G4 studies (A Contemporary, Prospective Study Evaluating Real-world Experience of Performance and Safety for the Next Generation of MitraClip Devices; NCT03502811). These studies were conducted to evaluate the real-world safety and effectiveness of the third-generation MitraClip NTR/XTR and fourth-generation MitraClip G4 Systems, respectively. Patients were enrolled across 91 centres in the USA, Canada, Europe, the Middle East, and Japan. From 2018 to 2022, a total of 2205 patients with symptomatic primary mitral regurgitation or secondary mitral regurgitation (SMR) were enrolled and treated according to regional indications for use. The EXPANDed studies were sponsored by Abbott and conducted in accordance with the latest Good Clinical Practice standards of the Declaration of Helsinki. Both studies received approval from local ethics committees and relevant regulatory authorities in the participating countries. All patients provided written informed consent. Additional details on the methodologies and outcomes from both studies have been published previously.^18–20^

To assess the impact of 30-day TR on outcomes, the analysis population was retrospectively identified from a pooled analysis of the EXPAND and EXPAND G4 studies and was limited to patients who met all of the following criteria:

- Underwent the MitraClip procedure with acute procedural success, defined as mitral regurgitation reduction to ≤2+ without death or mitral valve replacement at discharge; and
- Had an evaluable baseline TR of moderate-to-severe or severe, as assessed by the ECL in EXPANDed, which is representative of patients with severe, massive, and torrential TR according to the 5-grade TR scale by TVARC^21^ (referred to as ≥severe TR throughout); and
- Had an evaluable 30-day TR assessment by the ECL.

Patients were excluded from the analysis for any the following: MR and TR concomitant treatment during index procedure, prior tricuspid valve intervention, acute procedural failure with MR, follow-up treatment for TR following MTEER, baseline TR of moderate or less, no baseline TR assessment, and no 30-day TR assessment.

### Echocardiographic and clinical outcomes

Echocardiograms at all timepoints were evaluated by an independent ECL using a multiparametric integrative approach per the American Society of Echocardiography recommendations.^22^ Clinical events were adjudicated by an independent clinical events committee (CEC) through 30 days and site-reported through 1 year for EXPAND and were site-reported through 1 year for EXPAND G4. Transthoracic echocardiograms were performed in all patients at baseline, discharge, 30 days, and 12 months post-procedure.

Functional capacity, as assessed by the New York Heart Association (NYHA) Functional Class, and quality of life, as assessed by the Kansas City Cardiomyopathy Questionnaire-23 overall summary (KCCQ-OS) score, were performed at baseline, 30 days, and 12 months post-procedure. Safety and clinical outcomes, including all-cause mortality, cardiovascular mortality, stroke, myocardial infarction, heart failure hospitalizations (HFH), and mitral valve reintervention at the 1-year follow-up were also assessed.

### Statistical analysis

Baseline characteristics were summarized with means and standard deviations for continuous variables and proportions and numbers for categorical variables. Variables between TR progression groups were compared with a *t* test or Mann–Whitney *U* test for continuous variables and the χ^2^ or Fisher exact test for categorical variables. Bowker’s test was used to compare categorical variables paired across timepoints (e.g. TR severity, MR severity, and NYHA class). Significant differences in paired KCCQ-OS changes between baseline and one year were determined by Wilcoxon Signed rank test. For time-to-first event analyses, event rates were estimated by the Kaplan–Meier (KM) method and were compared using log-rank test. KM curves were landmarked to the 30-day TR assessment. Patients with HFH before the 30-day assessment were excluded from the HFH analysis. A univariate logistic regression was performed to identify possible associations of baseline characteristics with 30-day TR reduction. These characteristics included sex, STS replacement and repair score, prior HFH, atrial fibrillation, renal failure, prior myocardial infarction, Cardiac resynchronization therapy (CRT)/CRT-defibrillator (CRT-D)/implantable cardiac defibrillator (ICD) or permanent pacemaker, MR aetiology, TR, LV ejection fraction (LVEF), baseline and 30-day changes in LV dimensions (LV end-systolic and end-diastolic diameter [LVESD/LVEDD], LV end-systolic and end-diastolic volume [LVESV/LVEDV]), anteroposterior diastolic annular dimension (APDAD), baseline and 30-day changes in systolic pulmonary artery pressure, and mild or less MR at discharge. Associations were summarized with odds ratios (OR) and 95% confidential intervals (CI). A two-sided *P*-value <.05 was considered statistically significant for all tests. All statistical analyses were performed with SAS version 9.3 (SAS Institute, Cary, NC).

## Results

### Patient characteristics

Of the 2205 patients who enrolled in the study and underwent a MitraClip procedure, 160 patients had acute procedural success with the MitraClip device, had severe or greater TR at baseline, an evaluable TR at 30 days, and no prior, concomitant, or follow-up tricuspid valve procedure. Of these 160 patients, 116 (73%) showed improvement in TR to ≤moderate at 30 days, while 44 (28%) continued to have ≥severe TR (Supplementary Figure S1). Patients were excluded for having a prior or concomitant tricuspid valve procedure (*n* = 50) and acute procedural failure (*n* = 121). For patients who achieved acute procedural success with MitraClip, those who underwent TR treatment following the index procedure (*n* = 28), had a baseline TR ≤moderate (*n* = 1452), did not have an evaluable baseline TR assessment (*n* = 375), or did not have an evaluable 30-day TR assessment (*n* = 42) were excluded from the analysis.

Baseline and echocardiographic characteristics for the subgroups are presented in *Table 1*. Demographics, risk assessments, and comorbidities were similar between the groups, except for atrial fibrillation (AF), which had a lower prevalence in patients with 30-day TR ≤ moderate compared with those with 30-day TR ≥severe (67.8% vs 88.6%, *P* = .009). The aetiology of MR was similar between the groups. Regarding echocardiographic measurements, patients with 30-day TR ≤ moderate had larger LV dimensions and lower LVEF. A shorter hospital stay was observed in patients with 30-day TR ≤ moderate compared with those with 30-day TR ≥ severe (4.6 ± 5.6 vs 6.6 ± 6.1 days, *P* = .002), but the procedural (80.5 ± 43.0 vs 85.6 ± 44.6, *P* = .5) and device time (47.0 ± 42.8 vs 55.8 ± 40.3, *P* = .09) was similar. More than half of the patients in both groups (59% and 64%) had only 1 clip implanted (Supplementary Table S1). Heart failure medication usage is reported in Supplementary Table S2. At baseline, more patients with 30-day TR ≤ moderate reported antiarrhythmic medication use (25.9% vs 9.1%, *P* = .02). A majority of patients were on diuretics at baseline. Lower, but not statistically significant, usage was observed in patients with 30-day TR ≤moderate compared with 30-day TR ≥severe (80.2% vs 90.9%, *P* = .10).

**Table 1:** Baseline characteristics of patients stratified by the degree of tricuspid regurgitation at 30 days

Baseline characteristic	30-day TR ≤ Moderate (*N* = 116)	30-day TR ≥ Severe (*N* = 44)	*P*-value
Age	78.3 ± 10.0 (116)	80.7 ± 6.4 (44)	.14
Female	52.6% (61)	50.0% (22)	.77
STS replacement score (%)	8.5 ± 6.9 (78)	9.1 ± 4.9 (24)	.66
STS repair score (%)	6.6 ± 6.6 (88)	7.2 ± 5.1 (27)	.64
Prior heart failure hospitalization within 1 year	55.0% (60)	56.1% (23)	.91
Atrial fibrillation	67.8% (78)	88.6% (39)	.009
Renal failure	28.7% (33)	34.1% (15)	.51
Prior myocardial infarction	21.1% (24)	16.3% (7)	.50
CRT/CRT-D/ICD/Permanent Pacemaker	37.9% (44)	31.8% (14)	.47
NYHA class III or IV	71.3% (82)	84.1% (37)	.10
KCCQ-OS score	43.4 ± 24.0 (110)	47.5 ± 26.6 (43)	.36
Secondary MR aetiology	51.9% (56)	51.5% (17)	.48
LVEF (%)	49.2 ± 17.6 (99)	55.9 ± 14.1 (41)	.07
LVEDV (ml)	137.5 ± 73.4 (99)	107.9 ± 44.8 (41)	.01
LVESV (ml)	76.4 ± 64.9 (99)	49.8 ± 33.3 (41)	.02
LVEDD (cm)	5.5 ± .9 (111)	5.2 ± 0.8 (42)	.05
LVESD (cm)	4.2 ± 1.3 (111)	3.7 ± 0.9 (40)	.04
Systolic pulmonary artery pressure (mmHg)	54.2 ± 17.6 (109)	56.1 ± 14.8 (42)	.53

Data presented as mean ± standard deviation (*n*) or % (*n*).

CRT, cardiac resynchronization therapy; CRT-D, cardiac resynchronization therapy-defibrillator; ICD, implantable cardiac defibrillator; KCCQ-OS, Kansas City Cardiomyopathy Questionnaire Overall Summary; LVEDD, left ventricular end-diastolic diameter; LVEDV, left ventricular end-diastolic volume; LVESD, left ventricular end-systolic diameter; LVESV, left ventricular end-systolic volume; LVEF, left ventricular ejection fraction; NYHA, New York Heart Association; MR, mitral regurgitation; STS, Society of Thoracic Surgeons; TR, tricuspid regurgitation.

### Mitral and tricuspid regurgitation through one year

**Figure 1:**
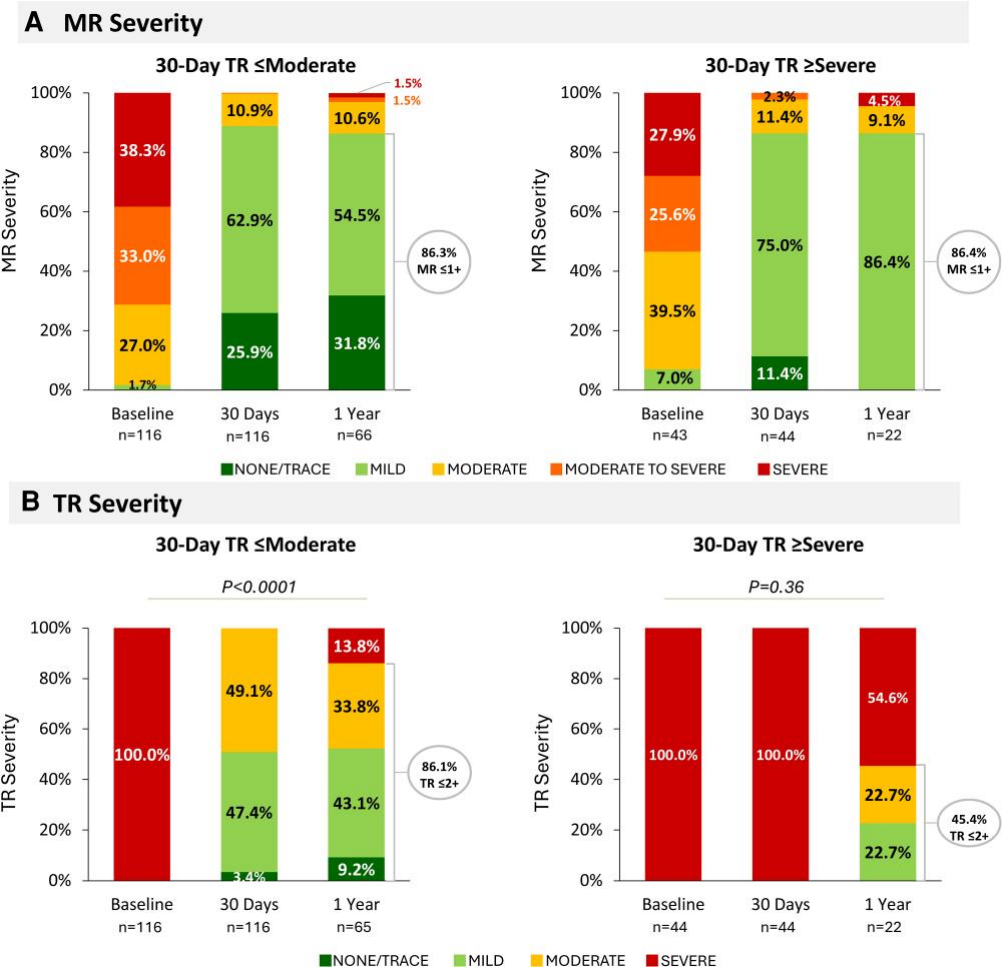
Mitral regurgitation (MR) and tricuspid regurgitation (TR) through 1 year. MR severity in patients with 30-day TR ≤ moderate and 30-day TR ≥ severe both improved significantly at 30 days and sustained through 1 year (*A*). Most patients with 30-day TR ≤ moderate maintained this grade at 1-year follow-up, and 45.4% patients with TR ≥ severe at 30 days improved to ≤moderate at 1 year (*B*). Severe TR includes moderate-to-severe and severe TR as assessed by the echo core lab in EXPANDed and are representative of the severe, massive, and torrential grades on the 5-grade TR scale by TVARC (Hahn RT, et al. *Ann Thoracic Surg*. 2023;116(5):908–932.)

MR grades significantly improved from baseline to 30 days in both groups and were sustained through 1 year, with 86.3% and 86.4% of patients achieving MR grades ≤ mild, respectively (*Figure 1A*). Among patients with 30-day TR ≤ moderate, 86.1% maintained a TR grade of ≤ moderate at one year. In contrast, 45.4% of patients with 30-day TR ≥ severe experienced a reduction in TR grade to ≤ moderate by one year (*Figure 1B*). From baseline to 30 days, there was a significant reduction in pulmonary artery systolic pressure (PASP) in patients with 30-day TR ≤moderate (Δ−8.4 ± 14.8 [86]; *P* < .0001, paired *t*-test) that was not observed in patients with ≥severe TR at 30 days (Δ−3.2 ± 9.8 [25]; *P* = .12, paired *t*-test, Supplementary Table S3).

### Clinical outcomes by 30-day tricuspid regurgitation

**Figure 2:**
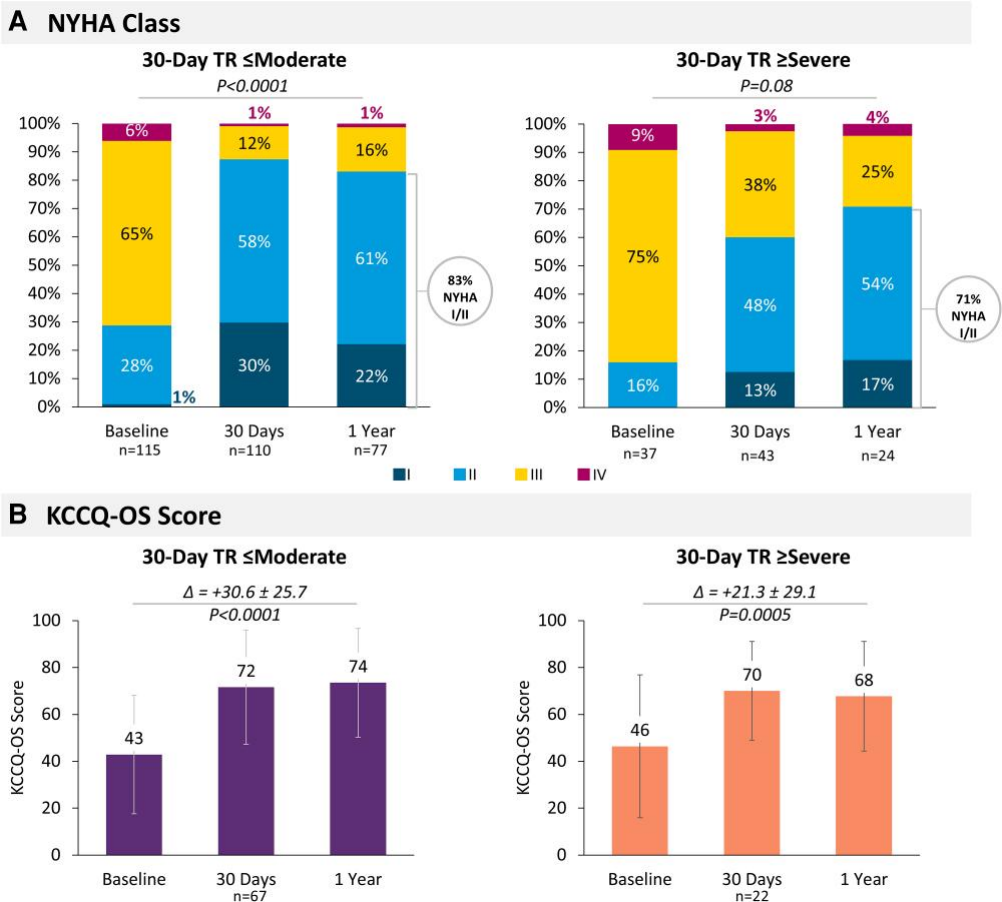
Functional capacity and quality of life improvement by 30-day tricuspid regurgitation (TR) severity. A significant improvement of NYHA functional class (*A*) and an improved quality of life according to KCCQ-OS score (paired analysis, *B*) was shown for both groups at 1 year. NYHA, New York Heart Association; KCCQ-OS, Kansas City Cardiomyopathy Questionnaire-Overall Summary

A significant improvement in functional status was observed in patients with improved TR at 30 days (*P* < .001), with a greater proportion of patients in NYHA Class I or II at 1 year (83%) from baseline (29%). Patients with 30-day TR ≥ severe also experienced similar improvement to NYHA Class I or II at 1 year compared with patients with 30-day TR ≤ moderate (*P* = .15), although less pronounced (71% NYHA I/II at 1 year vs 16% at baseline, *P* = .08, *Figure 2A*). Both groups showed a similar significant increase in KCCQ-OS scores after the MitraClip procedure (*P* = .16), with quality-of-life improvement sustained through 1 year (30-day TR ≤ moderate: Δ ± SD = 30.6 ± 25.7, *P* < .0001; 30-day TR ≥ severe: Δ ± SD = 21.3 ± 29.1, *P* = .0005). Patients with improved TR at 30 days experienced a numerically greater improvement in KCCQ-OS score compared with baseline (*Figure 2B*). Most patients continued to be on diuretics at 30 days. There were no significant differences in heart failure or diuretic medications between groups at 30 days (Supplementary Table S2).

**Figure 3:**
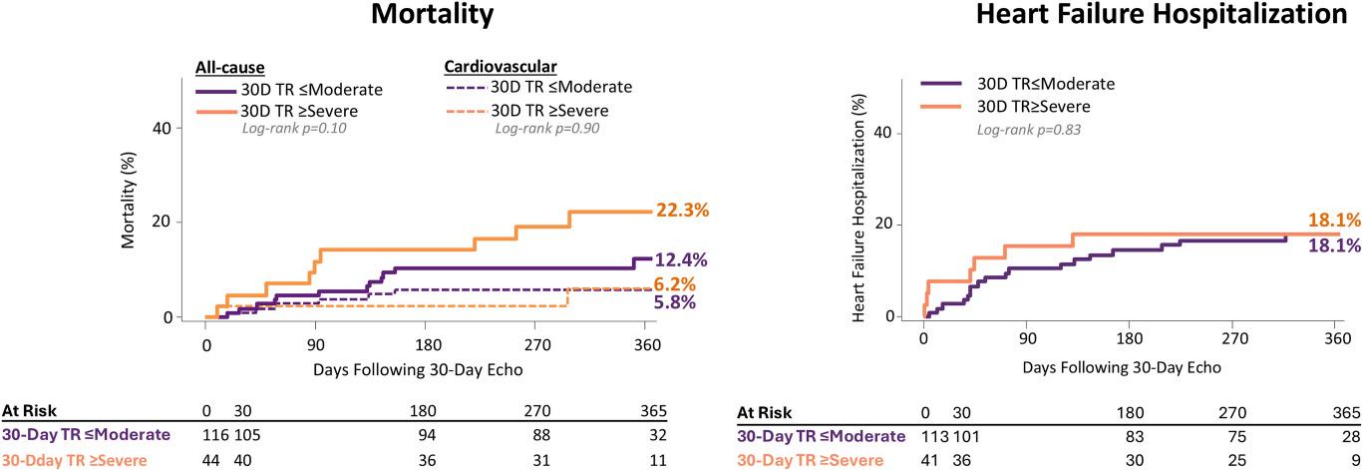
Kaplan–Meier estimates of all-cause mortality and heart failure hospitalization. At one year, patients with 30-day TR ≤ moderate exhibited a two-fold higher absolute all-cause mortality rate compared with those with TR ≥ severe [HR 1.92 (0.77, 4.79), *P* = .16]. However, rates of heart failure hospitalization were similar between the two groups. Kaplan–Meier curves were landmarked to the 30-day echo used to define the TR severity

Patients with 30-day TR ≥ severe had a numerically, but not significantly, higher KM estimated all-cause mortality rate at 1 year compared with those with TR improvement (22.3% vs 12.4%, *P* = .10), while the cardiovascular mortality rate was similar between the groups (6.2% vs 5.8%, *P* = .90) in a landmarked analysis at the 30-day echo (*Figure 3*). The one-year KM estimated heart failure hospitalization rates, landmarked at the 30-day echo, were comparable (18.1% vs 18.1%, *P* = .83). Other major adverse events from baseline through 1 year—including stroke, myocardial infarction, and mitral valve reintervention—were similarly low (<2.7%) across both groups (*Table 2*, *Figure 3*).

**Table 2:** Major adverse events through 1 year by 30-day tricuspid regurgitation severity

1-year major adverse events	30-day TR ≤Moderate (*N* = 116)	30-day TR ≥Severe (*N* = 44)	*P* value
All-cause mortality^a^	10.3% (11)	22.3% (9)	.08
Cardiovascular mortality^a^	5.7% (6)	6.2% (2)	.88
Stroke	2.7% (3)	0.0% (0)	.56
Myocardial infarction	0.9% (1)	0.0% (0)	1.00
Mitral valve surgical reintervention	0.0% (0)	0.0% (0)	1.00

^a^1-year mortality presented as Kaplan–Meier estimates with log-rank comparison *P*-values. Events counted from index MitraClip procedure. One death occurred after 365 days in the 30-day TR ≤Moderate group that was included in the 1-year Kaplan–Meier estimate of mortality landmarked to the 30-day echo. Data presented as % (*n*).

TR, tricuspid regurgitation.

### Factors associated with 30-day TR improvement

**Figure 4:**
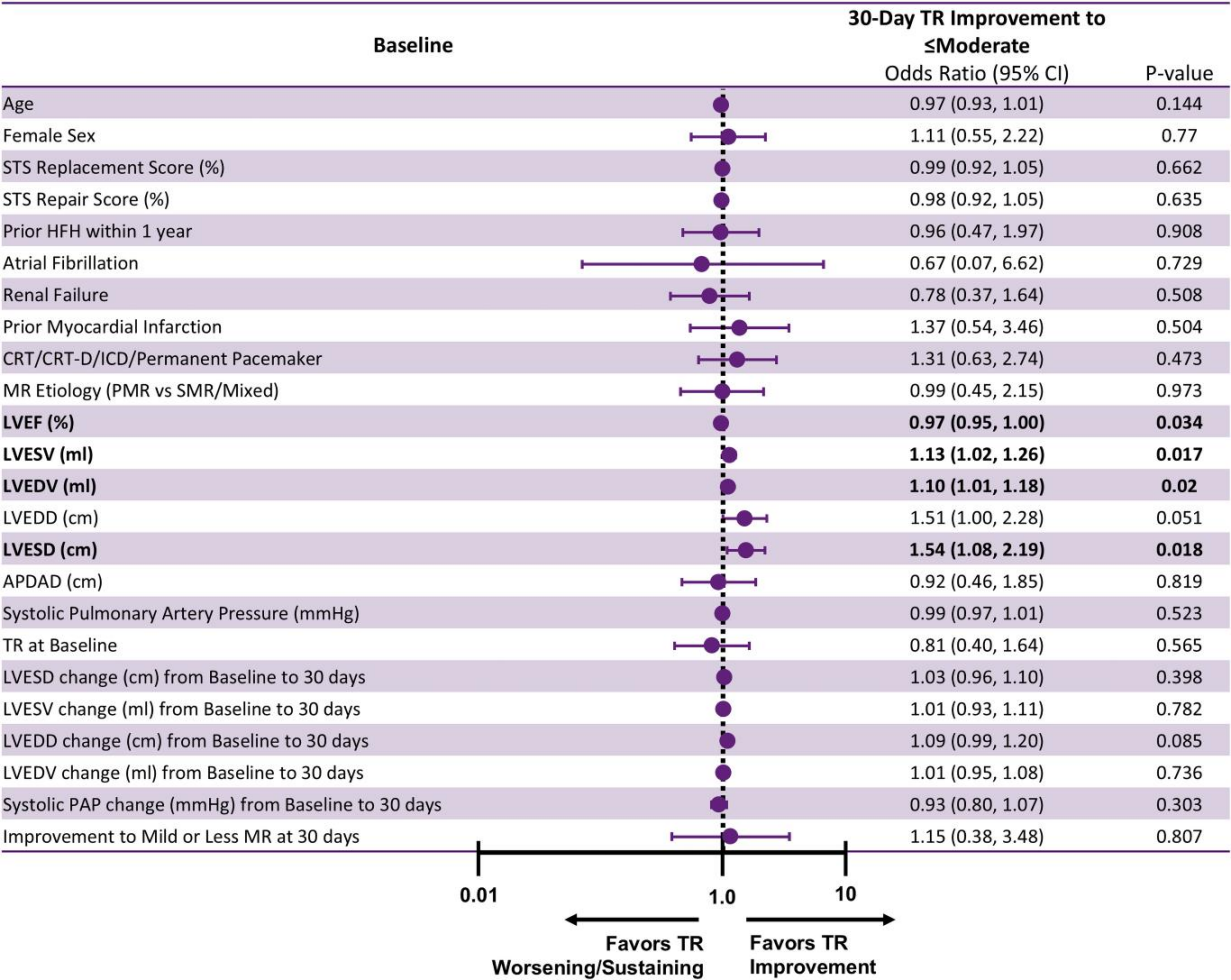
Associations with TR improvement at 30 days. Univariate logistic regression adjusted for age, sex, and baseline MR was used to identify potential factors associated with TR Improvement at 30 days. APDAD, anterior posterior diastolic annular dimension; CRT, cardiac resynchronization therapy; CRT-D, cardiac resynchronization therapy-defibrillator; ICD, implantable cardiac defibrillator; HFH, heart failure hospitalization; LVEDD, left ventricular end-diastolic diameter; LVEDV, left ventricular end-diastolic volume; LVEF, left ventricular ejection fraction; LVESD, left ventricular end-systolic diameter; LVESV, left ventricular end-systolic volume; MR, mitral regurgitation; PAP, pulmonary artery pressure; STS, Society of Thoracic Surgeons; TR, tricuspid regurgitation

A univariate logistic regression model adjusted for age, sex, and baseline MR was used to identify potential factors associated with 30-day TR improvement following MTEER (*Figure 4*). TR improvement at 30 days was associated with larger LVESV [OR (95% CI) = 1.13 (1.00–1.29), *P* = .0471] and changes in LVEDD from baseline to discharge [1.12 (1.01, 1.25), *P* = .0384].

## Discussion

This analysis represents the largest evaluation with ECL-assessed measures to date of the natural progression of TR following MTEER in patients with both MR and TR, using data from the EXPANDed studies. Key findings include (i) those who achieved TR reduction to ≤ moderate experienced greater improvements in quality of life, NYHA functional class, and lower 1-year mortality rates, although not statistically different, compared with patients with ≥severe TR at 30 days and (ii) baseline factors associated with 30-day TR improvement included larger LV dimensions and lower LVEF, suggesting that patients with left-heart dominant dysfunction may derive greater TR reduction after MTEER, possibly due to a correction of left heart volume overload.

Significant TR is frequently observed in patients undergoing MTEER, with reported prevalence ranging from 16% and 58% depending on population characteristics. Severe TR has been consistently associated with worse clinical outcomes.^15,23,24^ Recent retrospective studies have demonstrated varying rates of TR improvement after MTEER. The Northwell TEER registry, constructed from four prospectively maintained databases from high-volume centres, reported a TR reduction in 42% of patients at 1 month.^15^ Gröger et al. reported a TR decrease of at least one grade within 12 months in 30.2% of patients in a 300-subject single-centre registry.^16^ A retrospective analysis of 503 SMR patients from 13 European centres showed a ≥ 1 degree of TR improvement in 35% of patients after MTEER at a median follow-up of 79 days, with those experiencing TR improvement having significantly lower all-cause mortality at 3 years (29.6% vs 42.3%).^17^ Within a cohort of 311 patients with moderate or greater TR at baseline, 41% showed TR improvement, which was associated with better event-free survival regarding post-procedural HFH.^25^

In our study, we observed a higher rate of TR improvement compared with previous studies. The higher TR improvement rate after MTEER may be attributed to the more significant and sustained MR reduction observed in the EXPANDed studies.^18,19^ Patients in EXPAND G4 were treated with the fourth-generation MitraClip devices, which have been shown to exhibit more durable MR reduction.^18–20^ In our cohort, 86% of patients in both groups had an MR ≤ 1+ at 1 year.

Consistent with prior research,^26^ our analysis also showed that patients with 30-day ≥severe TR had higher all-cause mortality, although the conclusions are limited by sample size and limited follow-up duration. Cardiovascular mortality rates were similar between groups, indicating that all-cause mortality was likely driven by non-cardiovascular causes. Patients with 30-day TR reduction experienced comparatively greater symptom relief, albeit not statistically different, as evidenced by significant improvement in NYHA functional class (from baseline, *P* < .0001) and KCCQ-OS score (Δ = + 30.6 ± 25.7, *P* < .0001) than those with 30-day TR of severe or greater. These results support the clinical benefit of TR improvement and reinforce the role of TR as a modifiable risk factor post-MTEER.

Previous studies have identified female sex, baseline MR grade, baseline TR grade, baseline left atrial diameter, MR reduction, AF and tricuspid annular diameter as predictors of TR improvement after MTEER.^15–17,25–27^ Similarly, we also observed a higher prevalence of AF in patients with sustained ≥severe TR at 30 days. This underscores the importance of integrated AF management in MTEER candidates with severe TR, as AF may perpetuate tricuspid annular dilation and leaflet malcoaptation, independent of LV function, and patients with AF may also represent those with atrial functional MR, a phenotype associated with residual TR after MTEER.^27^ These patients may benefit from more aggressive rhythm control and timely valve intervention.^28^ In addition, we found that patients with TR reduction exhibited more pronounced LV dilation and lower LVEF at baseline, suggesting that MTEER may be particularly effective in those with left-heart dominant pathophysiology. Left-sided heart failure medications have been shown to improve RV function,^29^ and in a synergistic manner, the immediate reduction in TR may further halt the vicious cycle of pulmonary hypertension and right ventricular remodelling. Conversely, patients with relatively mild LV dilation may have TR driven more by right-sided dysfunction, and may not experience as significant of benefits from MR reduction alone. The results herein should be viewed as hypothesis generating and further investigation is needed to evaluate optimal treatment plans for these patients, particularly those with primarily right-sided disease.

### Limitations

Several limitations should be considered. Transcatheter tricuspid interventions were commercially unavailable in the USA and other regions during the years the EXPANDed studies were conducted. The EXPANDed studies were conducted to primarily assess changes in left-heart measures following MTEER in a patient population with left heart disease; therefore, data on right-heart structure and function were limited were limited and categorization into atrial vs ventricular valvular disease could not be performed. Medication usage in the EXPANDed studies was limited to the proportion of patients taking medication from baseline to 30 days and dosages were not collected. Clinical events in EXPAND study underwent an independent adjudication by CEC; those in EXPAND G4 were site-reported. This methodological difference could introduce variability in event classification. However, since most reported clinical outcomes were objective (e.g. mortality, stroke, MI), the potential for discrepancies between site-reported and CEC-adjudicated events were likely minimal. The relatively small sample size and limited follow-up duration may reduce the ability to detect significant associations, particularly mortality, and the ability to perform multivariable analysis. Patients who withdrew or died before the 30-day echo were excluded from the analysis, which may result in an overestimation of treatment efficacy. These results should be considered hypothesis-generating, and longer-term follow up with larger cohorts is needed to validate these findings.

## Conclusions

This analysis represents the largest assessment of the natural progression of TR after MTEER in patients with concomitant MR and TR using ECL-assessed measures from post-market EXPANDed studies. Patients with improved 30-day TR to ≤moderate had larger improvements in quality of life and lower 1-year mortality rates, though not statistically significant, compared with those with ≥severe 30-day TR. Associations with TR improvement suggest that those with dilated LVs at baseline may experience more significant relief from TR after MTEER, possibly due to relief of left-sided volume overload. Further investigation is needed to evaluate optimal treatment plans for these patients with concomitant MR and TR, particularly those with primarily right-sided dysfunction.

### Impact on daily practice

This study highlights that MTEER in patients with concurrent MR and severe TR yields significant TR improvement in two thirds of patients, particularly in those with left-sided dominant dysfunction. TR improvements after MTEER are associated with significant symptom relief and quality of life improvement at 1 year. Right-sided dysfunction should be suspected in patients with severe concomitant TR but relatively mild LV dilation. A comprehensive evaluation and follow-up TR assessment should be conducted to determine the optimal treatment plan for this subgroup of patients.

## Supplementary Material

xvag108_Supplementary_Data

## Declarations

### Data Availability

No data were generated or analysed for this manuscript.

### Pre-registered Clinical Trial Number

None supplied.
