# Supplementary material for: Prognosis of tricuspid regurgitation after mitral transcatheter edge-to-edge repair: the EXPANDed studies
Source: ESC Heart Fail. 2026 Apr 16;13(3):xvag108. doi: 10.1093/eschf/xvag108 (PMC13202460; doi:10.1093/eschf/xvag108)
Supplement: xvag108_Supplementary_Data [file xvag108_supplementary_data.zip › Supplemental Figure_Legends.docx]

**Supplemental Figure S1. Analysis population.**

A total of 2205 patients were enrolled in the EXPANDed study. Of these, 27 patients with concomitant MR and TR treatment and 23 patients with previous TV procedures were excluded. Among the 2034 patients who achieved acute procedural success with MitraClip implantation, 28 patients who underwent follow-up TR treatment were further excluded. After identifying 202 patients with concomitant severe baseline TR, a 30-day assessment of TR was performed. An additional 42 patients were excluded due to unevaluable TR assessments. Finally, 116 patients were categorized with 30-day TR severity ≤moderate, and 44 with TR ≥ severe at 30 days. APS, acute procedural success; MR, mitral regurgitation; TR, tricuspid regurgitation; TV, tricuspid valve.

**Supplemental Figure S2. Sankey diagram of TR in a paired analysis at baseline, 30 days, and 1 year from patients with 30-day TR ≤Moderate and with 30-day TR ≥Severe**. Severe TR encompasses moderate-to-severe and severe TR as assessed by the echo core lab in EXPANDed and are representative of the severe, massive, and torrential grades on the 5-grade TR scale by TVARC (Hahn RT, et al. Ann Thoracic Surg. 2023;116(5):908-932.)
