# Supplementary material for: Prognosis of tricuspid regurgitation after mitral transcatheter edge-to-edge repair: the EXPANDed studies
Source: ESC Heart Fail. 2026 Apr 16;13(3):xvag108. doi: 10.1093/eschf/xvag108 (PMC13202460; doi:10.1093/eschf/xvag108)
Supplement: xvag108_Supplementary_Data [file xvag108_supplementary_data.zip › Supplemental Table S.docx]

**Supplemental Table S1. Procedural details by 30-day tricuspid regurgitation grade.**

|  | 30-Day TR≤Moderate  (N=116) | 30-Day TR ≥Severe  (N=44) | P-value |
| --- | --- | --- | --- |
| Length of hospital stay (days) | 4.6 ± 5.6 | 6.6 ± 6.1 | 0.002 |
| Procedure time (minutes) | 80.5 ± 43.0 | 85.6 ± 44.6 | 0.50 |
| Device time (minutes) | 47.0 ± 42.8 | 55.8 ± 40.3 | 0.09 |
| Mean number of clips implanted |  |  | 0.91 |
| 1 clip | 58.6% (68) | 63.6% (28) |  |
| 2 clips | 36.2% (42) | 31.8% (14) |  |
| 3 clips | 5.2% (6) | 4.5% (2) |  |

Data presented as mean±standard deviation (n) or % (n). TR, tricuspid regurgitation.

**Supplemental Table S2. Changes in medications from Baseline to 30 Days.**

|  | 30-Day TR ≤Moderate  (N=116) | | 30-Day TR ≥Severe  (N=44) | |
| --- | --- | --- | --- | --- |
| **Medication** | **Baseline** | **30 Days** | **Baseline** | **30 Days** |
| **ACE-Inhibitors (ACE-I)** | **21.6 (25)** | **23.3 (27)** | **34.1 (15)** | **34.1 (15)** |
| **Aldosterone Antagonists** | **26.7 (31)** | **32.8 (38)** | **20.5 (9)** | **29.5 (13)** |
| **Angiotensin Receptor Blockers** | **25.0 (29)** | **22.4 (26)** | **22.7 (10)** | **18.2 (8)** |
| **Antiarrhythmic** | **25.9 (30)** | **23.3 (27)** | **9.1 (4)** | **11.4 (5)** |
| **Beta-Blockers** | **75.0 (87)** | **70.7 (82)** | **81.8 (36)** | **84.1 (37)** |
| **Entresto** | **12.1 (14)** | **12.1 (14)** | **4.5 (2)** | **4.5 (2)** |
| **Diuretics** | **80.2 (93)** | **82.8 (96)** | **90.9 (40)** | **86.4 (38)** |

**Data presented as % (n).**

**Supplemental Table S3. Changes in Pulmonary Artery Systolic Pressure through 1 year**

|  | Baseline | 30 Days | 1 Year |
| --- | --- | --- | --- |
| **30 day TR ≤ moderate** | **54.2 ± 17.6 (109)** | **45.9 ± 11.6 (93)** | **43.8 ± 13.2 (41)** |
| **30-day TR ≥ severe** | **56.1 ± 14.8 (42)** | **53 ± 16.1 (27)** | **50.8 ± 18.1 (15)** |

**TR, tricuspid regurgitation; Data presented as mean±standard deviation (n).**
