# Supplementary figures and images for: Prognosis of tricuspid regurgitation after mitral transcatheter edge-to-edge repair: the EXPANDed studies
Source: ESC Heart Fail. 2026 Apr 16;13(3):xvag108. doi: 10.1093/eschf/xvag108 (PMC13202460; doi:10.1093/eschf/xvag108)

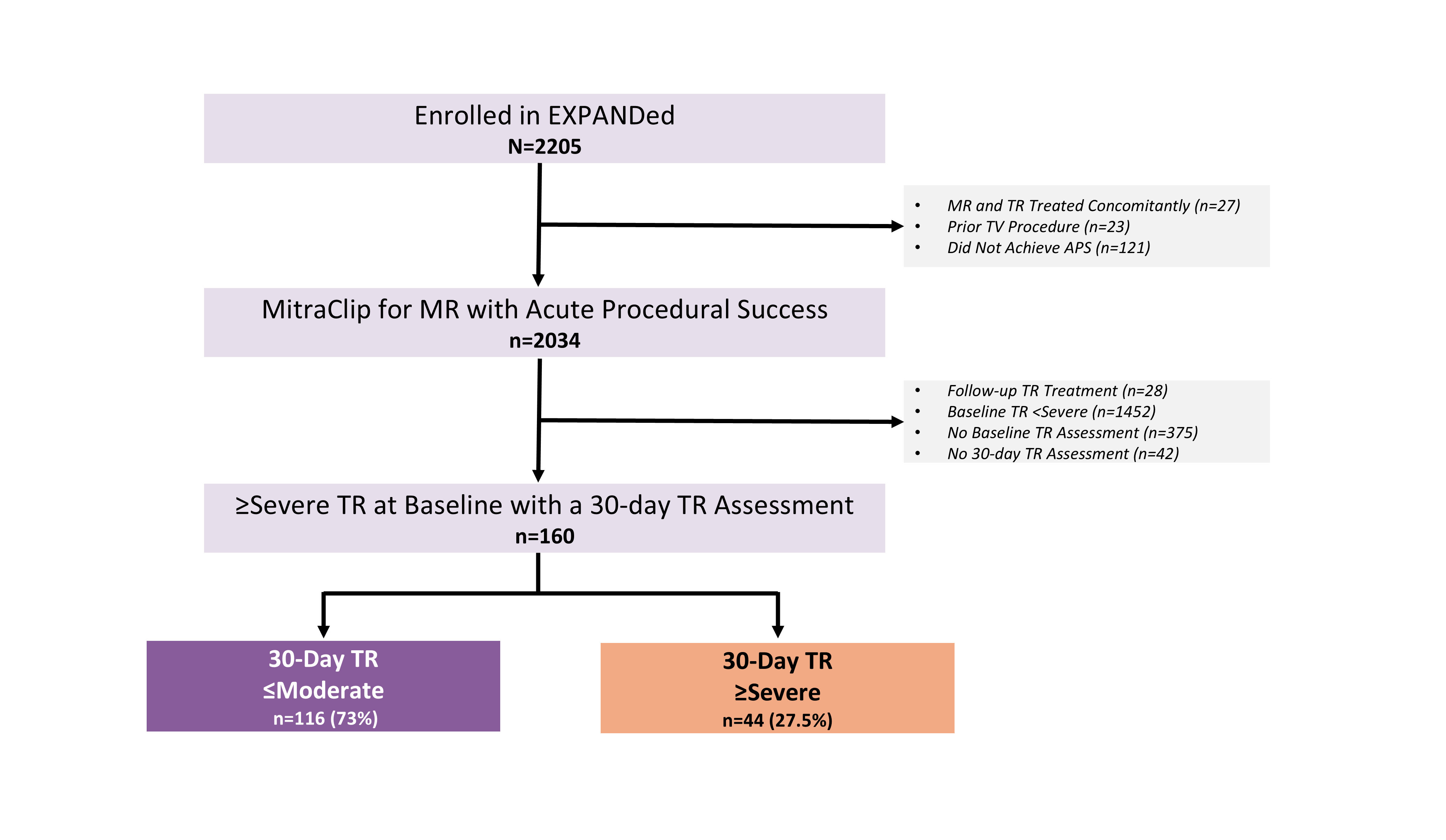

Supplement: xvag108_Supplementary_Data [file xvag108_supplementary_data.zip › Supplemental Figure S1.tif]

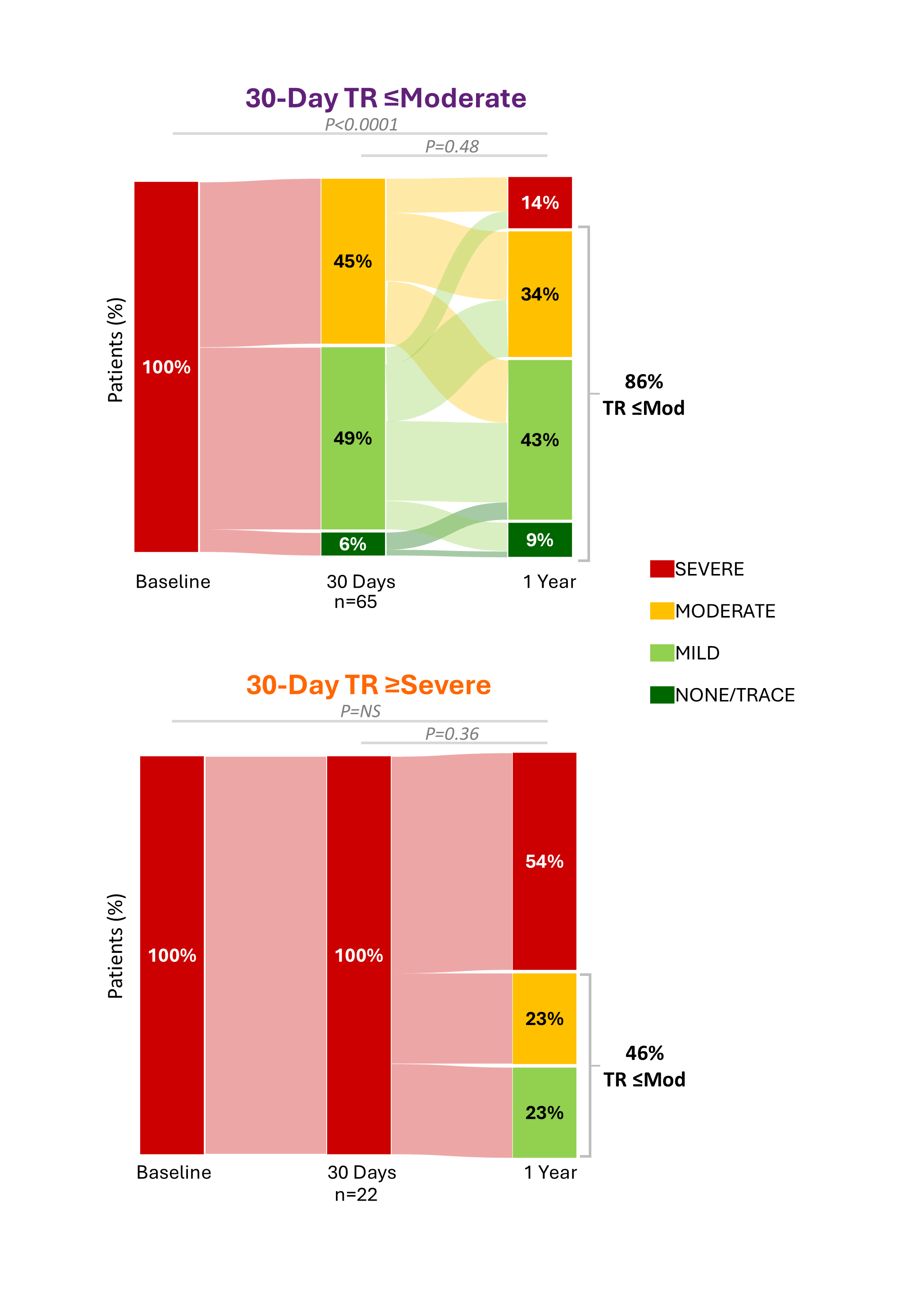

Supplement: xvag108_Supplementary_Data [file xvag108_supplementary_data.zip › Supplemental Figure S2.tif]
